# Trehalose-Based Eye Drops Preserve Viability and Functionality of Cultured Human Corneal Epithelial Cells during Desiccation

**DOI:** 10.1155/2014/292139

**Published:** 2014-06-08

**Authors:** Aneta Hill-Bator, Marta Misiuk-Hojło, Krzysztof Marycz, Jakub Grzesiak

**Affiliations:** ^1^Department of Ophthalmology, Medical University Wroclaw, Borowska 213, 50-556 Wroclaw, Poland; ^2^Electron Microscopy Laboratory, University of Environmental and Life Sciences in Wroclaw, Kozuchowska 5b, 51-631 Wroclaw, Poland

## Abstract

This paper presents the evaluation of cytoprotective ability of trehalose-based eye drops in comparison with commercially available preparations during the experimental desiccation of cultured human corneal epithelial cells. Cultured human corneal epithelial cells (hCEC) underwent incubation with 7 different, commercially available medicaments used commonly in dry eye syndrome treatment, followed by desiccation trial performed on air under the flow hood for 5, 15, 30, and 45 minutes. Cell viability was quantified by live/dead fluorescent assay, while the presence of apoptotic cells was estimated by immunofluorescent staining for active caspase 3 protein. The preservation of membrane functions was evaluated using neutral red staining, while the preservation of proper morphology and phenotype was determined by fluorescent staining for actin filaments, nuclei, and p63 protein. The trehalose-based eye drops showed the highest efficiency in prevention of cell death from desiccation; moreover, this preparation preserved the normal cellular morphology, functions of cell membrane, and proliferative activity more effectively than other tested medicaments.

## 1. Introduction

Dry eye syndrome is a wide spread disease, caused by environmental influence or organism's function disorders. These different grounds allowed dividing it on three types: simple dry eyes (SDE), autoimmune positive dry eyes (ADE), and Sjogren's syndrome (SS) [1]. Inadequate quantity of tears or pathologic composition of them puts up the superficial part of eyes on adverse conditions. Dry corneal epithelium is exposed to rubbing, leading to scar formation, irritation, and inflammation. It leads to various occurrences, including squamous metaplasia, corneal neovascularization, or thinning, among others [2]. In the tears of patients suffering dry eye syndrome, the presence of proinflammatory cytokines, such as interleukin-6 and tumor necrosis factor-alpha, is very common [3]. In case of ADE and SS, also the autoaggressive immunoglobulins causing additional inflammatory and allergic reactions can be found [4]. Different grounds limit the general therapy to symptomatic treatment of disorder's outcomes. Therefore, lack or deficiency of tears can be settled by lubricating the cornea with external preparations. There are many types of commercially available medicaments composed of various substances, which prevent the loss of moistness, promoting the wound healing or decreasing the inflammation. However, the mechanisms of their action are often connected with their water retention ability, like in hyaluronian- or carboxymethylcellulose-based preparations. The last investigations revealed a disaccharide named trehalose, which has been described as a protective factor for various cells [5]. It is a nonreducing disaccharide present in many prokaryotic and eukaryotic organisms, which plays a role as the source of energy and carbon. In plants and yeasts it also occurs as the signaling molecule with the ability of cell protection, mainly from desiccation, dehydration, extreme temperatures, or oxidation [6]. Experiment performed by Guo et al. showed that human primary fibroblasts transfected with adenovirus vector for induction of trehalose synthesis could be maintained alive in the dry state for up to five days [7]. Therefore, this molecule could be found as potential medicament in dry eye syndrome. In the first experiments, applications of trehalose solution prevented the corneal epithelial cells from dying after drying during* in vitro* experiment [8]. As the natural cause of things, dry eye syndrome-treating preparations containing the trehalose occurred for commercial use. Therefore, we decided to compare the trehalose-based eye drops available on European market with six other, commonly used in clinical practice in a laboratory experiment. The efficiency in preservation of the viability and function of human corneal epithelial cells (hCEC) was evaluated during desiccation trials performed using* in vitro* cell culture model. Cells cultured as monolayer were dried by putting on air without any medium or liquid, pretreated before with medicaments encountered to this research, with respect to normal saline, and not treated control [9]. Cells were then analyzed for vitality by live/dead fluorescent staining and active caspase 3 detection and functionality by p63 protein detection and neutral red staining.

Tested preparations differed in composition and in the mechanism of protection, so substantial differences in results should be expected. Besides the trehalose sample, preparations were based on common viscoelastic substances known for keeping the moistness, like polyvinyl alcohol, hyaluronic acid, or methylcellulose. All results revealed trehalose-based medicament as the most effective in preventing the negative outcomes in hCEC cultures resulting from desiccation trials.

## 2. Materials and Methods

### 2.1. Cell Culture

Cell lines and all reagents used in this research were obtained from Life Technologies. 2 vials of frozen human corneal epithelial cells (5 × 10^5^ cells/vial) were thawed and plated in T-25 flasks in Serum-Free Corneal Epithelium Medium at a concentration of 1.5 × 10^5^ per T-25 flask. Prior to plating, the tissue culture flasks were coated with collagen for increasing the cell attachment. So prepared cultures were maintained in 37°C/5%CO_2_/95% humidity until cells reached almost full confluency (5–7 days). Culture medium was changed every 48 hours. Confluent cultures were trypsinized with TrypLE Express and the cells were split to collagen-precoated, chambered coverslips at a concentration of 1 × 10^4^ cells/cm². After cells adhered properly, they underwent experiments.

### 2.2. Eye Drops Application

The eye drops preparations for comparative analysis with Thealoz were chosen based on their commercial availability and popularity in patients. Additionally, they were chosen for different composition and preservatives included. The trade names, producers, and general compositions are shown in Table 1.

Epithelial cell monolayers were washed with warm normal saline and covered with seven kinds of preparations for five minutes (*n* = 3) in 37°C/5%CO_2_/95% humidity. Control samples were treated with normal saline or not treated. After the incubation, preparations were discarded and the coverslips were put on air under the fume hood without any liquid for 5, 15, 30, and 45 minutes. In the next step they underwent evaluation of viability and functionality.

### 2.3. Live/Dead Assay

After the desiccation trial, cultures were stained with live/dead assay kit. Briefly, they were washed with PBS and incubated with fluorescent dyes (red propidium iodide indicated dead cells and green calcein-AM live cells) for 15 minutes. After that, dyes were discarded, layers washed with PBS and covered with Fluoromount (Sigma) to avoid bleaching. After staining, cells were examined under AxioObserver A1 inverted, fluorescent microscope (Zeiss). The number of live and dead cells was evaluated by manual counting of every repeat of every sample. The results were averaged and analyzed for statistical significance of differences between the Thealoz and other preparations using one-way analysis of variance (one-way ANOVA test), with Bonferroni posttest determining the statistical significance of differences between all other preparations (GraphPad Prism, GraphPad Software).

For detection of apoptotic cells, immunocytochemical staining for active caspase 3 was applied. Prior to fixation, cells were allowed to give a response on desiccation trial by incubating them in medium and 37°C/5%CO_2_/95% humidity for one hour. After incubation, cells were washed with PBS and fixed with 3.7% cold paraformaldehyde for 10 minutes. After fixation, cells were triple washed in PBS with 1% FBS and permeabilized with Triton X-100 (0.1% in PBS) for 15 minutes at room temperature. In the next step, cells were triple washed and incubated with primary antiactive caspase 3 antibody produced in rabbit for one hour in 37°C/5%CO_2_/95% humidity, followed by triple washing and incubation with secondary anti-rabbit IgG-atto-594 antibody for one hour in 37°C/5%CO_2_/95% humidity. After triple washing, cells were incubated with phalloidin-atto-488 dye for visualization of f-actin and DAPI for nuclei staining. Cells were triple washed, covered with fluoromount, and observed in fluorescent, inverted microscope. The documentation was made using Cannon PowerShot Camera.

### 2.4. Functionality Test

For functionality test, samples desiccated for 15 minutes were chosen, in view of optimal cell number obtained from viability assay. Cells were evaluated for the presence of p63 protein by immunofluorescent staining. After desiccation trials, cells were washed in PBS, covered with medium, and incubated in 37°C/5%CO_2_/95% humidity for one hour. In the next step, cells were washed with PBS, fixed with 3.7% cold paraformaldehyde for 10 minutes at room temperature, triple washed in PBS, and permeabilized with Triton X-100 (0.1% in PBS) for 15 minutes. After triple washing, cells were incubated with primary anti-p63 antibody produced in rabbit for one hour at 37°C/5%CO_2_/95% humidity, followed by triple washing and incubation with secondary anti-rabbit IgG-atto 594 for one hour at 37°C/5%CO_2_/95% humidity. After triple washing, cells were incubated with phalloidin-atto-488 dye for visualization of f-actin and DAPI for nuclei staining. Cells were triple washed, covered with fluoromount, and observed in fluorescent, inverted microscope. The documentation was made using digital Cannon PowerShot Camera. Cells were evaluated for p63 presence or absence and localization of detected protein.

For evaluation of cell membrane function, neutral red staining was applied. After desiccation trials, cells were washed in PBS and incubated in medium at 37°C/5%CO_2_/95% humidity for one hour. In the next step cells were incubated with neutral red dye for 15 minutes, washed triple times in PBS, and observed and documented using inverted microscope and digital camera. Cells were evaluated for visible dye uptake, which indicates the proper cellular membrane function.

## 3. Results

### 3.1. Live/Dead Assay

**Figure 1:**
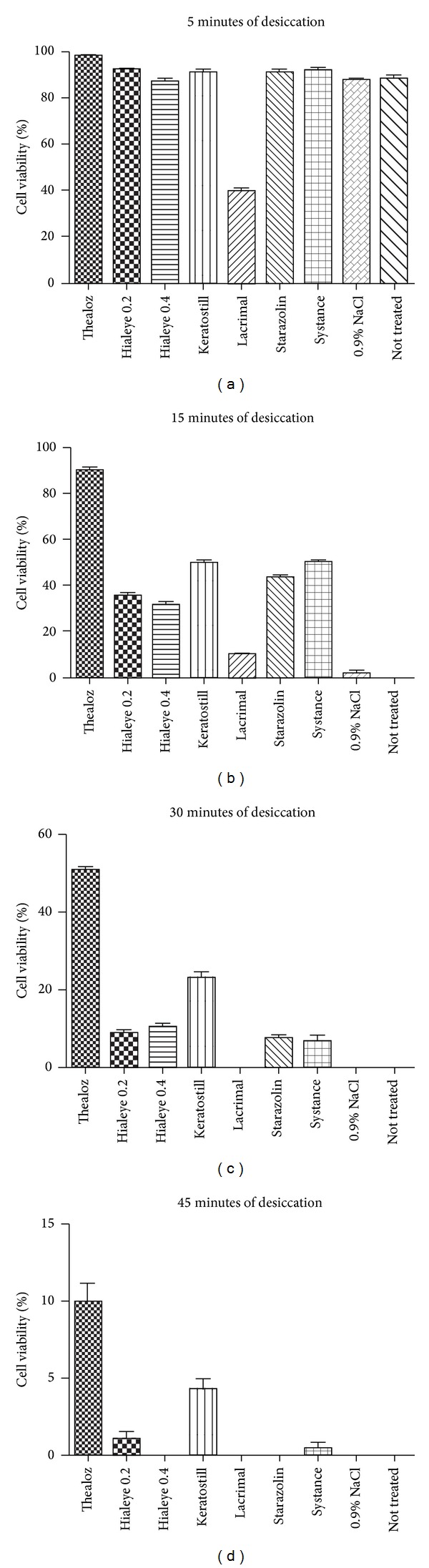
The results of live/dead assay made after 5, 15, 30, and 45 of desiccation, shown as percentage of living cells with standard deviation marked.

After application of different eye drops and desiccation trials, substantial differences in cell viability could be noticed between particular preparations. After 5 minutes of desiccation, similar results from 6 preparations were obtained. The samples treated with Thealoz showed the statistically higher viable cell number (*P* < 0.01) than 0.9% NaCl control-treated samples. Lacrimal samples showed the lowest cell number prominently different from other results. After 15 minutes of desiccation, Thealoz samples showed the highest effectiveness in preventing cell death (*P* < 0.01), while the remaining preparations could be ordered from more to less efficient: Keratostill, Starazolin, Systane, Hialeye 0.2 and Hialeye 0.4, and Lacrimal as the least efficient, respectively; statistically no significant differences were obtained between two pairs of preparation: Keratostill and Systane and between Hialeye 0.2 and 0.4 (*P* > 0.05). After 30 minutes of desiccation, Thealoz was still the most effective (*P* < 0.01). The second most effective preparation was determined as Keratostill, while the Hialeye 0.2 and 0.4, Starazolin, and Systane showed similar results (*P* > 0.05), slightly less effective than Keratostill. In Lacrimal samples no viable cell could be noticed. After 45 minutes of desiccation, the only viable cells were detected in Thealoz and Keratostill samples. The difference between these two samples and 0.9% NaCl control was statistically significant (*P* < 0.01). Detailed results are shown in Table 2 and Figure 1.

**Figure 2:**
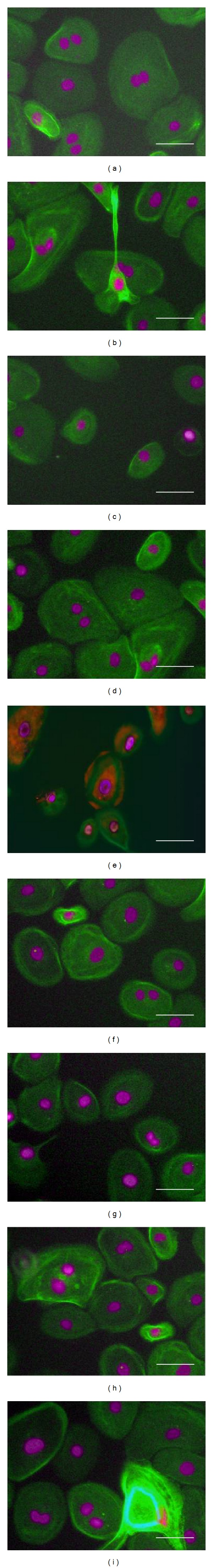
Immunocytochemical staining for active caspase 3 (red), actin (green), and nuclei (violet) in corneal epithelial cells after the treatment with tested preparations and 15 minutes of desiccation. Only in sample treated with Lacrimal (e) apoptotic cells were present; (a) Thealoz, (b) Hialeye 0.2, (c) Hialeye 0.4, (d) Keratostill, (e) Lacrimal, (f) Starazolin, (g) Systane, (h) 0.9% NaCl control, and (i) not treated control; magnification 200x; scale bar = 40 um.

Interestingly, the only apoptotic cells were detected in Lacrimal samples, with prominently visible active caspase 3 and characteristic changes in cellular morphology determined by actin filaments and in nuclei structure (Figure 2(e)).

### 3.2. Functionality Test

**Figure 3:**
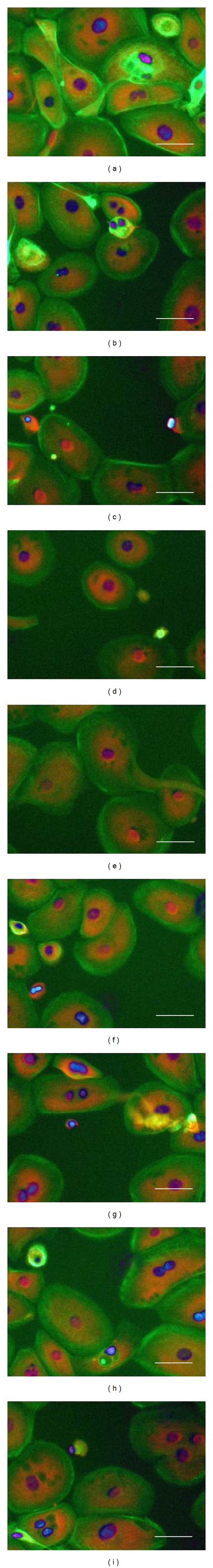
Immunocytochemical stainings for p63 (red), actin (green), and nuclei (blue/violet) in corneal epithelial cells after the treatment with tested preparations and 15 minutes of desiccation. The presence of p63 protein in nuclei and cytoplasm is prominent in all tested cells; (a) Thealoz, (b) Hialeye 0.2, (c) Hialeye 0.4, (d) Keratostill, (e) Lacrimal, (f) Starazolin, (g) Systane, (h) 0.9% NaCl control, and (i) not treated control; magnification 200x; scale bar = 40 um.

Microscopic observations revealed cells with characteristic, epithelial morphology with actin organized smoothly without visible filaments, keeping cells in round shape. Evaluation of p63 protein presence showed its proper localization in every sample also in 0.9% NaCl and not treated control (Figures 3(a)–3(i)).

**Figure 4:**
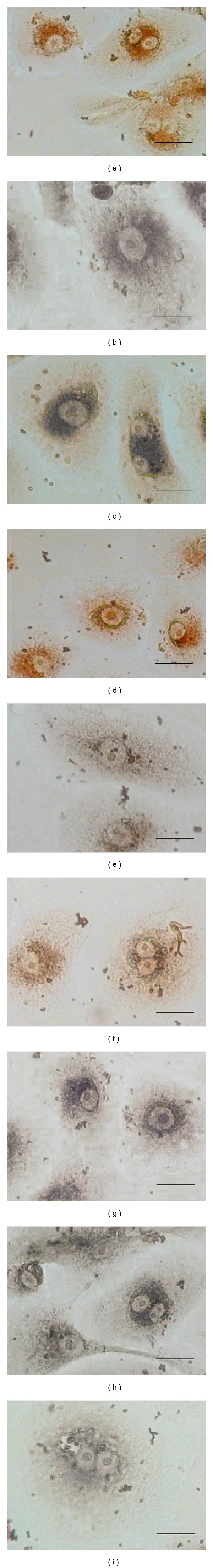
The neutral red staining of corneal epithelial cells after treatment with tested preparations and 15 minutes of desiccation. Only in cells treated with Thealoz (a) and Keratostill (d) a dye uptake may be observed. In the remaining samples only slight or no reaction was noticed. (a) Thealoz, (b) Hialeye 0.2, (c) Hialeye 0.4, (d) Keratostill, (e) Lacrimal, (f) Starazolin, (g) Systane, (h) 0.9% NaCl control, and (i) not treated control; magnification 400x; scale bar = 20 um.

However, the neutral red staining showed different effects of applied eye drops on cellular membrane functionality and endosomal transportation system. In samples treated with Thealoz and Keratostill, the neutral red dye uptake was prominent (Figures 4(a) and 4(d), resp.). In the remaining samples, cells did not stain which indicated the loss of cellular membrane function (Figures 4(b)-4(c) and 4(e)–4(i)).

## 4. Discussion

Dry eye syndrome is a chronic disease, which can be induced by various factors. Therefore, any general treatment has not been elaborated yet, although there are preparations that help in keeping the eye in moistness. The most of available preparations are based on hyaluronic acid (HA) or carboxymethylcellulose (CMC). The protection mechanism of hyaluronian-based eye drops is connected with HA's natural lubricating ability, by both binding the water and adsorbing to the ocular surface without allergic reactions [10]. CMC is the main compound of artificial tear drops. It is a high-molecular-weight polysaccharide with mucoadhesive properties, which allow the preparation for prolonged residence time on the ocular surface. It has been shown that application of CMC onto damaged cornea significantly enhances the wound healing process, although the mechanism of this action remains unclear [11]. Due to the discovery of the trehalose's antidehydrating nature, it was concluded that it may help in dry eye syndrome. This disaccharide not only protects cells from desiccation, but also is able to preserve the cell membrane and membrane proteins from deactivation or denaturation, as it was shown by others [6]. We state that this feature of trehalose is the most important advantage, resulting in highest cell survival ratio and maintenance of cell membrane function. In our research, one preparation was based on CMC derivate-the hydroxypropyl methylcellulose (Keratostill). The live/dead assay showed Keratostill, after the Thealoz, as the most effective in cell death prevention. Additionally, these particular eye drops together with Thealoz were able to preserve the function of cellular membrane and retain the intracellular transportation system, which was indicated by neutral red staining. Thus we conclude that this preparation has also the capacity to protect cell membranes during desiccation; however, the mechanism is still uncovered. On the other hand, preparations containing other active agents resulted in less cell survival ratio and even induced apoptosis (Lacrimal). This preparation is based on polyvinyl alcohol, commonly used in artificial tears, which possess high oxygen barrier properties [12, 13]. Basing on the fact that epithelial cells obtain the oxygen directly from atmospheric air not from blood, we conclude that these outstanding results of Lacrimal could be caused by the lack of oxygen. While the Thealoz is preservatives-free, we conclude that these significant differences in cytoprotective ability between preparations may also result from the presence of particular preservatives, like benzalkonium chloride (BAK, in Hialeye and Lacrimal) or polidronium chloride (Polyquad, PQ in Systane). As it was shown by others, BAK induces cell death and arrests cell growth even at low concentration [14, 15]. The cytotoxic effect of PQ can also be noticed, although it seems to be much less prominent when compared to BAK [16]. Interestingly, the preparation Hialeye 0.2 was more effective than Hialeye 0.4 only after 5-minute desiccation trial, which has the concentration of HA two times greater. We suspect that in Hialeye 0.4, besides the higher concentration of HA there is also a higher concentration of BAK, although such information was not provided by the producer. Finally, the expression of p63 protein was not altered in any sample. This molecule is connected with proliferation and stratification of CEC progenitors [17, 18], so we conclude that none of preparation has the negative influence on normal corneal regeneration.

In conclusions, the preparation based on trehalose showed the highest effectiveness in preventing cell death from desiccation and in keeping the function of cellular membranes, in comparison to other eye drops. Additionally, we confirmed that different preservatives have different negative effect on cell function and viability, as it was shown by others. Therefore, the trehalose-based, preservative-free eye drops are the advanced medicament for dry eye syndrome disease.

## Figures and Tables

**Table 1:** Trademarks, producers, and components of tested eye drops. Data based on information provided by the producer.

Preparation (producer)	Composition
Thealoz (Thea)	3% trehalose; sodium chloride; trometamol; hydrochloric acid; water for injections
Hialeye 0.2 (Blaufarma)	0.2% sodium hyaluronate; disodium phosphate; sodium dihydro orthophosphate; sodium chloride; sodium edetate; benzalkonium chloride; water for injections
Hialeye 0.4 (Blaufarma)	0.4% sodium hyaluronate; disodium phosphate; sodium dihydro orthophosphate; sodium chloride; sodium edetate; benzalkonium chloride; water for injections
Keratostill (Bruschettini S.R.I.)	0.3% hydroxypropyl methylcellulose; dexpanthenol; EDTA; dibasic sodium phosphate; deionized water; cetrimide 0.01%
Lacrimal (WZF Polfa)	Polyvinyl alcohol; 12-water disodium phosphate; sodium dihydrophosphate monohydrate; sodium chloride; benzalkonium chloride; deionized water
Starazolin hydrobalance (Polpharma OTC)	0.1% sodium hyaluronate; sodium chloride; sodium orthophosphate; sodium tetraborate stabilized with phosphonic acid
Systane (Alcon)	Polyethylene glycol 400; polypropylene glycol; hydroxy propylene guar; sorbitol; aminomethyl propanol; boric acid; potassium chloride; sodium chloride; 0.001% polidronium chloride

**Table:** (a)

	5 min					15 min				
	Mean [%]	St. dev.	Max	Min	*P* value	Mean [%]	St. dev.	Max	Min	*P* value
Thealoz	98.77	0.45	99.2	98.3	<0.01	90.65	2.05	92.1	89.2	<0.01
Hialeye 0.2	92.63	0.75	93.4	91.9	<0.05	35.1	2.26	36.7	33.5	<0.01
Hialeye 0.4	87.37	2.34	89.1	84.7	ns	32.1	0.28	32.3	31.9	<0.01
Keratostill	92	1.1	93.1	90.9	ns	50.05	3.04	52.2	47.9	<0.01
Lacrimal	40.1	1.34	41.2	38.6	<0.01	10.25	1.48	11.3	9.2	<0.01
Starazolin	91.53	1.62	93.3	90.1	ns	44.05	0.35	44.3	43.8	<0.01
Systane	92.9	0.36	93.2	92.5	<0.05	50	0.28	50.2	49.8	<0.01
0.9% NaCl	88.43	1.17	89.7	87.4		2.4	1.13	3.2	1.6	
Not treated	89.63	1.12	90.6	88.4		0	0	0	0	

**Table:** (b)

	30 min					45 min				
	Mean [%]	St. dev.	Max	Min	*P* value	Mean [%]	St. dev.	Max	Min	*P* value
Thealoz	51.75	1.48	52.8	50.7	<0.01	10.25	2.76	12.2	8.3	<0.01
Hialeye 0.2	8.15	0.35	8.4	7.9	<0.01	0.6	0.85	1.2	0	ns
Hialeye 0.4	11.2	1.55	12.3	10.1	<0.01	0	0	0	0	ns
Keratostill	23.5	2.83	25.5	21.5	<0.01	4.45	1.77	5.7	3.2	<0.01
Lacrimal	0	0	0	0	ns	0	0	0	0	ns
Starazolin	7.85	0.35	8.1	7.6	<0.01	0	0	0	0	ns
Systane	6.85	3.32	9.2	4.5	<0.01	0.6	0.85	1.2	0	ns
0.9% NaCl	0	0	0	0		0	0	0	0	
Not treated	0	0	0	0		0	0	0	0	
